# Interest in Humans: Comparisons between Riding School Lesson Equids and Assisted-Intervention Equids

**DOI:** 10.3390/ani11092533

**Published:** 2021-08-28

**Authors:** Noémie Lerch, Francesca Cirulli, Céline Rochais, Clémence Lesimple, Estelle Guilbaud, Laura Contalbrigo, Marta Borgi, Marine Grandgeorge, Martine Hausberger

**Affiliations:** 1 University Rennes, Normandie University, CNRS, EthoS (Éthologie animale et humaine)–UMR 6552, F-35380 Paimpont, France; celine.rochais@gmail.com (C.R.); lesimple.c@gmail.com (C.L.); est.guilbaud@gmail.com (E.G.); marine.grandgeorge@univ-rennes1.fr (M.G.); martine.hausberger@univ-rennes1.fr (M.H.); 2 Center for Behavioral Sciences and Mental Health, Istituto Superiore di Sanità, Viale Regina Elena 299, I-00161 Rome, Italy; francesca.cirulli@iss.it (F.C.); marta.borgi@iss.it (M.B.); 3 Italian National Reference Centre for Animal Assisted Interventions, Istituto Zooprofilattico Sperimentale delle Venezie, Viale Dell’Università 10, 35020 Legnaro (Padua), Italy; lcontalbrigo@izsvenezie.it

**Keywords:** animal-assisted interventions, apathy, *Equus caballus*, human–horse relationship, individual characteristics

## Abstract

**Simple Summary:**

Very little is known about the impact of equine-assisted interventions on equids’ perception of humans. Different factors can influence human–horse relationships: animal characteristics, daily interactions with the caretakers, and working and living conditions. In this study, 172 equids working in equine-assisted interventions, ‘classical’ riding school lessons, or both were submitted to a standardised human–horse relationship test in order to test if EAI had an impact on the equid reactions to humans. The possible influence of intrinsic (age, sex, type) or other extrinsic factors (housing and feeding conditions) was also considered. The results showed that the number (more than the type) of experimenter-directed behaviours varied significantly between individuals and that the activity was the most important factor of influence: Equids working in riding school lessons performed more interactive behaviours than those working in equine-assisted interventions or having mixed activity. Other factors such as daily hay quantity, the horses’ age, and sex also influenced secondarily the horse’s motivation to interact, although no interaction was found between factors. These results suggest that equine-assisted interventions do influence horses’ perception of humans outside work. Further studies are needed in order to understand the processes involved.

**Abstract:**

Little is known about the impact of equine-assisted interventions (EAI) on equids’ perception of humans. In this study 172 equids, living in 12 riding centres, were submitted to a standardised human–horse relationship test: the motionless person test. Age, sex, type (horse/pony), housing, and feeding conditions of subjects were recorded. Overall, 17 equids worked in EAI, 95 in riding school lessons (RS), and 60 in both (EAI-RS). There were high inter-individual variations in the number of interactive behaviours directed towards the experimenter: negative binomial general linear models showed that activity was the most important factor: RS equids performed more interactive behaviours than EAI (*p* = 0.039) and EAI-RS (*p* < 0.001) equids. Daily quantity of hay appeared as the second most important factor (equids with more than 3 kg interacted more than equids with less than 3 kg, *p* = 0.013). Individual characteristics were also important as horses interacted more than ponies (*p* = 0.009), geldings more than mares (*p* = 0.032), and 3–15-year-old equids more than equids over 15 years (*p* = 0.032). However, there was no interaction between factors. The lower number of interactive behaviours of EAI equids leads to different hypotheses—namely, selection on temperament, specific training, or compromised welfare (apathy). In any case, our results raised new lines of questions on EAI.

## 1. Introduction

Animal-assisted interventions (AAIs), are defined by the International Association of Human–Animal Interaction Organisations [1] as ‘goal oriented and structured interventions that intentionally include or incorporate animals in health, education and human services (e.g., social work) for the purpose of therapeutic gains in humans’, and have become increasingly popular [2]. AAIs use various animals [2], amongst which horses are one of the most frequently used species (e.g., for children with autism spectrum disorders) [3]. The effects of these equine-assisted interventions (EAIs) on humans have been widely studied [4,5,6], but studies focusing on the animals involved remain scarce. Two main opinions prevail concerning how equids perceive these activities. One common belief is that horses ‘sense’ the ‘needs’ of persons, adapt to them, and appreciate these activities [7,8] —an anthropocentric view according to McGreevy et al. [9], whereas other authors argue that horses may find it difficult to deal with persons with unusual behaviours or postures [10,11] or even that an AAI animal’s welfare could be affected negatively [12]. 

Importantly, EAI horses have to also deal with the same constraints in their daily life as other working horses, with potential spatial and social restrictions and/or inappropriate feeding conditions that can compromise their welfare [13,14,15,16,17]. Researchers also suggested that repeated influences of poor working conditions can affect horses’ overall chronic welfare state [18] and even develop learned helplessness, a state where they have lost their ability to react to negatively perceived stimuli [19]. The reasons can be that riding may influence the comfort of horses during riding (e.g., rider’s posture [20,21]; equipment [22,23]), leading to potential dorsal problems [24,25]. Dorsal pain may induce aggressive reactions in human–horse relationship tests [26] and affect the cognitive assessment of their environment [27]. Horses experiencing compromised welfare can also become unresponsive to their environment, including humans [28,29,30]. Improvement of feeding/housing conditions is associated with more positive reactions towards humans [31,32,33]. Moreover, behavioural differences can be observed early in life and show some stability, leading some authors to propose that motivation to interact (i.e., interest in humans) can be considered as an equine temperament trait [34,35,36,37,38]. Thus, differences in reactions towards humans have been found according to the type of equid (ponies or horses), breed, or sire [39,40,41,42,43].

However, the reactions of horses in human–horse relationship tests reflect primarily their perception, positive or negative, of humans ([44] for a review) as horses are able to generalise the relationship they have with their owner, caretaker, trainer, or rider to an unknown human [30,39,45,46]. In addition, human–horse interactions are also influenced at the time of interaction by human characteristics, such as the human’s posture, attentional and emotional state, or experience with horses [47,48,49].

The human–horse relationship (HHR) is a crucial part of AAI, and the equids chosen for these activities are supposed to be calm and gentle, although experimental tests have shown that this is not necessarily the case [50]. During EAI, equids can be confronted with persons who have unpredictable behaviours [51], attention problems, aggression or irritability [10], negative moods [52], or balance problems [10]. As an example, in another species working in assisted intervention, it has been shown that while guinea pigs are first attracted when confronted for the first time with a child with autism spectrum disorders, they rapidly express discomfort (indicated by reduced frequency of feeding and exploration) [53]. However, Merkies et al. [48] observed no particular modification of the behaviour of horses moving freely towards patients with post-traumatic stress disorder or neurotypical humans. Most studies performed during EAI sessions suggest that the activity per se or type of person has no particular impact [48,51,52,54,55].

To our knowledge, however, no study has investigated the impact of EAI on the perception equids have of humans in general, i.e., outside working sessions, that is, as a reflection of the chronic impact these sessions might have (and not as immediate effects). One study showed that type of work can influence the reactions of horses in HHR tests outside work [56] and the quality of their working conditions has been shown to affect the reactions of horses towards unknown experimenters [28,57]. In low-income countries, horses used for draught work were less responsive to human approaches than riding horses [57,58], and breeding horses were more positive towards humans than working horses [59]. Training and working conditions have a major impact on the perception of humans that horses build, and this is the result of a generalisation process [46,60,61].

Therefore, we hypothesised that EAI horses, as a result of the particularities of these activities, would have a different perception of humans from that of riding school lessons horses beyond potential additional intrinsic (horse/pony, age or sex) or extrinsic (conditions of life amongst which housing and feeding conditions) factors. Standardised HHR tests have proved useful to evaluate this perception objectively [44]. Thus, to test this hypothesis, we chose a commonly used test, the motionless person (MP) test, to examine human-directed behaviours that reflect horses’ perception of humans and their motivation to interact (i.e., ‘interest’).

We expected differences in the reaction to an unknown human between equids according to their activity. We predicted differences in the valence of the behaviours displayed and differences in the ‘interest’ of equids for the unknown humans measured with the number of human-directed behaviours. We also expected an impact of conditions of life (i.e., feeding and housing conditions) with possibly more negative behaviours towards humans or fewer reactions in suboptimal conditions than in better conditions. Lastly, we also expected an impact of equids’ individual characteristics such as sex, age, or breed. 

## 2. Materials and Methods

### 2.1. Ethical Statement

The experiments were carried out between 2009 and 2019 in accordance with the Directive 2010/63/UE of the European Parliament and the Council on the protection of animals used for scientific purposes. They complied with the current French laws related to animal experimentation (decree n°2013 ± 118 of 1 February 2013) and its five implementation orders (JO 7 February 2013, integrated into the Rural Code and the Code of maritime fishing under n° R. 214 ± 87 to n° R. 214–137). The experiments performed in this study were not within the scope of application of the European directive; thus, in accordance with this directive and the current French, Italian, and Irish laws, the following experiments did not require us to request authorisation. These experiments involved only behavioural observations and non-invasive interactions with the horses. The horses used in this research were not research animals. Animal husbandry and care were under the management of the riding school staff. The riding school managers gave the authors their informed consent for this study.

### 2.2. Subjects

The subjects of this study were 172 equids (93 mares, 79 geldings, aged 4 to 29 years old, mean = 14.3 ± 0.4) that lived and were tested in 12 different riding centres (4 Italian, 1 Irish, and 7 French centres). They were 91 ponies ( ≤ 148cm at withers) and 81 horses ( > 148cm at withers) (International Federation for Equestrian Sport) from various breeds but mostly unregistered (Table 1). At the time of the study, they had been in their facility and had been involved in the same working practices for at least one year. Overall, 17 equids worked only in equine-assisted interventions (EAIs), 95 in ‘classical’ riding school lessons (RS), and 60 in both activities (EAI-RS; Table 1). The proportion of each activity could not be obtained in all mixed centres, but in the centres where the information could be collected, the proportion was between 7% and 86% of EAI activities, mean ± SE = 40.5 ± 5.1%. Each equid included in the study worked closely with at least three different persons. All centres used negative reinforcement for training. EAI activities were mostly grooming, groundwork, lunging, and riding. It was addressed to people with disabilities such as motor disabilities, visual, hearing, or cognitive impairments, crippling diseases, or other health diseases linked to psychosocial risks or social problems (e.g., jail, dropping out of school). 

Equids were housed in either individual stalls (78%) (size between 6m ² and 25m ²) or group stalls (22%) (size between 5.3m ² and 11.7m ² per equid); 5% of equids had no bedding in their stall, 71.5% had straw, and 34.5% had wood shavings. Feeding practices varied between facilities. All horses were fed hay: less than 3 kg per day (19%), between 3 kg and 9 kg (37.8%), or more than 9 kg per day (43.2%); commercial pellets: none (25.9%), one (9.2%), two (45.4%), or three (19.5%) meals per day. Water was provided ad libitum in all facilities, mostly through automatic drinkers.

### 2.3. Experimental Test

This study included experiments conducted between November 2009 and May 2019. Four female experimenters aged 24 to 30 years (CL, CR, EG, and NL), trained by a senior author (MH) (until an inter-observer reliability kappa coefficient of 0.80 was reached for each behavior expressed by equids during the test), performed the tests. In accordance with the results of Merkies et al. [48] and suggestions of Nimer and Lundahl [62], the tests were performed by experimenters with neurotypical development who were experienced with horses but not familiar with our subjects. Each equid was tested once by only one person. Equids from the same riding school were all tested by the same person. The equid had never met the experimenter before the start of the test.

The motionless person (MP) test is a standardised test used in many studies on human–horse relationships, but the exact procedure can vary slightly between studies (review in [44]). The aim of this test is to assess the spontaneous reaction of equids to the mere presence of an unknown experimenter in its familiar home environment. Equids were tested in their own individual or group stall. When it was possible, subjects were tested individually in the stall; however, some equids (N = 31) had to be tested in groups. The experimenter chose a moment when the horse was feeding on the ground, facing towards the door to enter the stall. If the horses were tested in a group, the experimenter chose a time when they were at equal distance from the door. At the beginning of the test, the experimenter entered the stall and stood motionless with her back against the closed door with her arms by her side without interacting with the equid for five minutes.

The equid’s behaviours were recorded by the experimenter during the test using a voice recorder and a lapel microphone attached under the clothes. In addition, the test was filmed using a camera attached to the outside of the stalls (the animals had been accustomed to the camera on the previous days), in order to verify that all behaviours had been recorded.

Tests were conducted in calm conditions, at least 30 min before or after work sessions, between 8 a.m. and 6 p.m., and at least 30 minutes before or after feeding times, because behaviours change in this period [63].

### 2.4. Data Analyses

The occurrences of all behaviours directed towards the experimenter were recorded using focal continuous sampling [64] during the five minutes the MP test lasted. Recorded behaviours were divided into different categories: behaviours considered as positive towards humans were gazes, approaches, sniffs, licks, and nibbles with ears forwards, while behaviours considered negative towards humans were gazes, approaches, sniffs, licks, and nibbles with ears laid back, threats or actual bites, or kicks and avoidance (e.g., [45,46]). Invasive physical contacts (i.e., biting clothes, head rubbing, pushing with head, and jostling) were not included in the positive behaviours because they could not be separated from frustration behaviours [31]. Moreover, behaviours expressed with ears on the side or asymmetrical ears were considered as ‘other behaviours’ since their valence is unclear (see Table 2 for more details). Behaviours not directed towards the experimenter were not considered for this analysis. The total numbers of behaviours displayed by each equid towards the unknown human during the test were calculated. 

### 2.5. Statistical Analyses

Analyses were conducted using R software (version 3.5.0) [65]. The significance threshold was *p* = 0.05 and descriptive statistics were reported as means and standard error. The number of positive and negative behaviours towards humans during the test was compared between EAI horses and RS horses using Kruskal–Wallis tests, followed by Mann–Whitney tests as post hoc tests. 

Negative binomial generalised linear mixed-effects models (GLMM.nb) (goft package) [66] were used to understand which factors explained best the individual differences in the number of human-directed interactive behaviours and to check for potential interactions between factors. GLMM.nb are classical models for count data (not normally distributed) and are adapted to situations where the explanatory variables are not balanced [67]. A first GLMM.nb was made including all following factors as fixed effects: test modality (in a group or alone), equid’s characteristics such as sex (mare or gelding), age (categorised according to Burn et al. (2010): < 15 years old or > 15 years old) and type (pony or horse), as well as the conditions of life factors such as type of housing (individual stall, group stall), hay quantity per day (0–3 kg, 3–9 kg or more than 9 kg), number of pellet meals per day (none, one, two or three) and type of activity (EAI, RS-EAI, or RS). Experimenters and centres were used as random effects. We followed a three-step procedure to test and select the best-fitting model. As the first step, in order to test if random effects are important for the model, a selection, based on the Akaike information criterion, (AICc) was made. Five models were tested: with centre and experimenter as random effects, with centre and experimenter as nested random effects, only with centre, only with the experimenter, and without any random effect. The model with the lower AICc was kept [68]. As the second step, we applied an automatic procedure based on the AICc (Package MuMIn) [69] on the previously chosen model to select which fixed effect to include in the model. The procedure tested all possible combinations of fixed effects and ranked the worst to the best fitting model. This procedure ensures that only significant factors are kept in the model. As a third step, all possible interactions were tested from the model chosen in the second step. The AICc was recalculated, and the interaction was only retained in the model if the AICc decreased by more than 2 points; this procedure ensures that only significant interactions are kept in the model. Once the final model was selected, an inspection of fitted values residuals was conducted using the plotresid function in RVAideMemoire package [70] in order to assess the independence and homogeneity of variances of the models, and the normality of the model residuals was tested using a Shapiro test. The estimate was used to assess the order of importance of the factors. 

The first step of the model analyses revealed that the model was better without random effect (AICc: 1077.70, ΔAICc: 0.00) than with experimenter and centre (AICc: 1082.53, ΔAICc: 4.84), nested experimenter and centre (AICc: 1082.53, ΔAICc: 4.84), only experimenter (AICc: 1080.10, ΔAICc: 2.40), or only centre (AICc: 1080.92, ΔAICc: 3.22) as random effects. In the second step, the 256 possible models were tested, and significant factors appeared. The third step showed that there was no significant interaction; therefore, the best model, which was the model without interaction (AICc: 1066.5, ΔAICc: 0.00), was retained.

Finally, because the distribution of age was not comparable among the EAI, RS-EAI, and RS equids, the number of human-directed interactive behaviours was compared between EAI, RS-EAI, and RS equids over 15 years old and then between EAI, RS-EAI, and RS equids under 15 years old using Kruskal–Wallis tests, followed by Mann–Whitney tests as post hoc tests. 

## 3. Results

**Figure 1:**
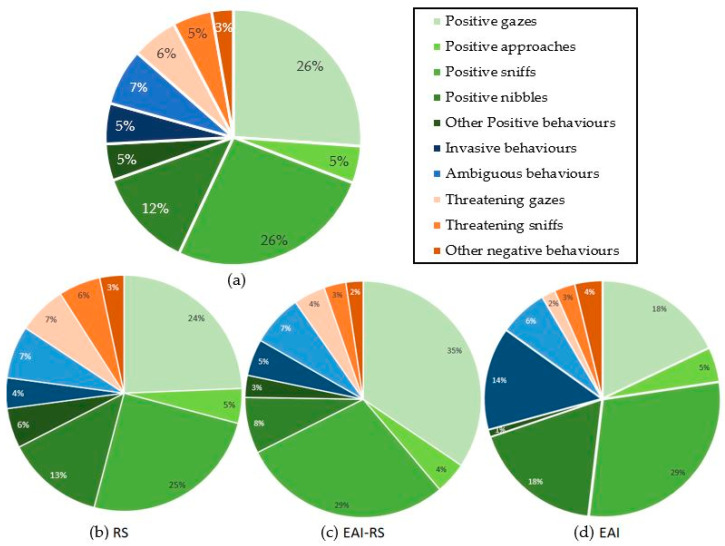
Proportion of behaviours expressed during the motionless person test by (**a**) all equids, (**b**) riding school lesson equids, (**c**) equids with mixed activities, and (**d**) assisted-intervention equids.

**Figure 2:**
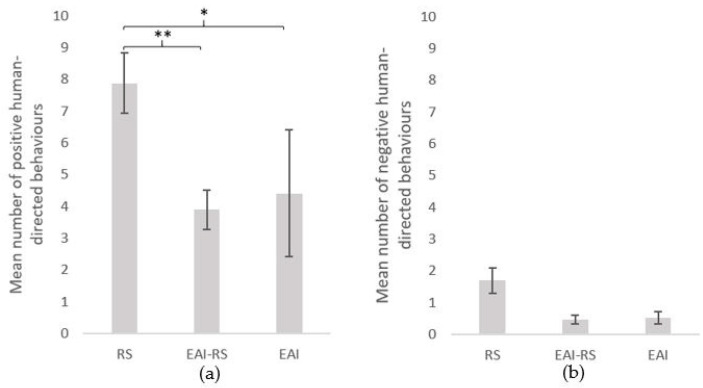
(**a**) Positive and (**b**) negative human-directed behaviours (mean ± SE) during the motionless person test. Tests followed by Mann–Whitney tests as post hoc tests. RS = riding school horses (N = 95), EAI = assisted-intervention horses (N = 17), EAI-RS = horse with mixed activity (N = 60). **: *p* < 0.01, *: *p* < 0.05.

The number of behaviours directed towards the experimenter during the 5 minutes of a test varied significantly between subjects (*n* = 172 equines; range of 0–51 behaviours; mean ± SE = 8.3 ± 0.8). During a test, 81% of the subjects displayed at least one positive behaviour and 27% at least one negative behaviour. Interestingly, 12% of the subjects tested did not express any behaviour towards the experimenter. Overall, the equids displayed a higher proportion of positive (ears forwards, sniffs: 26.1%; gazes: 26.0%; nibbles: 12.5%), than negative (ears backwards, gazes: 5.8%; sniffs: 4.9%) behaviours (Figure 1). Equids involved in riding school lessons (RS) displayed more positive behaviours (mean ± SE = 7.9 ± 0.9) than the equids involved in equine-assisted interventions whether it was only part of (EAI-RS) (mean ± SE positive = 3.9 ± 0.6) or their whole (EAI) (mean ± SE positive = 4.4 ± 2.0) activity (Kruskal–Wallis chi-squared = 12.4, df = 2, *p*-value = 0.002; Mann–Whitney tests as post hoc tests: RS vs. EAI-RS: W = 3716, *p*-value = 0.001; RS vs. EAI: W = 1074.5, *p*-value = 0.030; EAI-RS vs. EAI: W = 531.5, *p*-value = 0.793) (Figure 2). No significant difference was observed for the negative behaviours (mean ± SE: RS = 1.7 ± 0.4; EAI-RS = 0.5 ± 0.1; EAI = 0.5 ± 0.2) (Kruskal–Wallis chi-squared = 2.9, df = 2, *p*-value = 0.235) (Figure 2).

Since the most variable aspect was the total number of interactive behaviours, we tested further the possible factors of influence on this parameter. The comparison between the different GLM models revealed that five out of the eight factors tested had a significant influence: age, sex, type of equid (pony or horse), the quantity of hay, and type of activity (EAI, EAI-RS, or RS) (AICc: 1066.5, ΔAICc: 0.00), whereas single versus group housing (1068.0, ΔAICc: 1.5), individual versus group testing (AICc: 1070.4, ΔAICc: 3.9), and the number of pellet meals per day (AICc: 1071.0, ΔAICc: 4.5) did not seem to influence the number of behaviours towards the experimenter. There was no interaction between factors, the best model was the model without interaction (AICc: 1066.5, ΔAICc: 0.00). 

**Figure 3:**
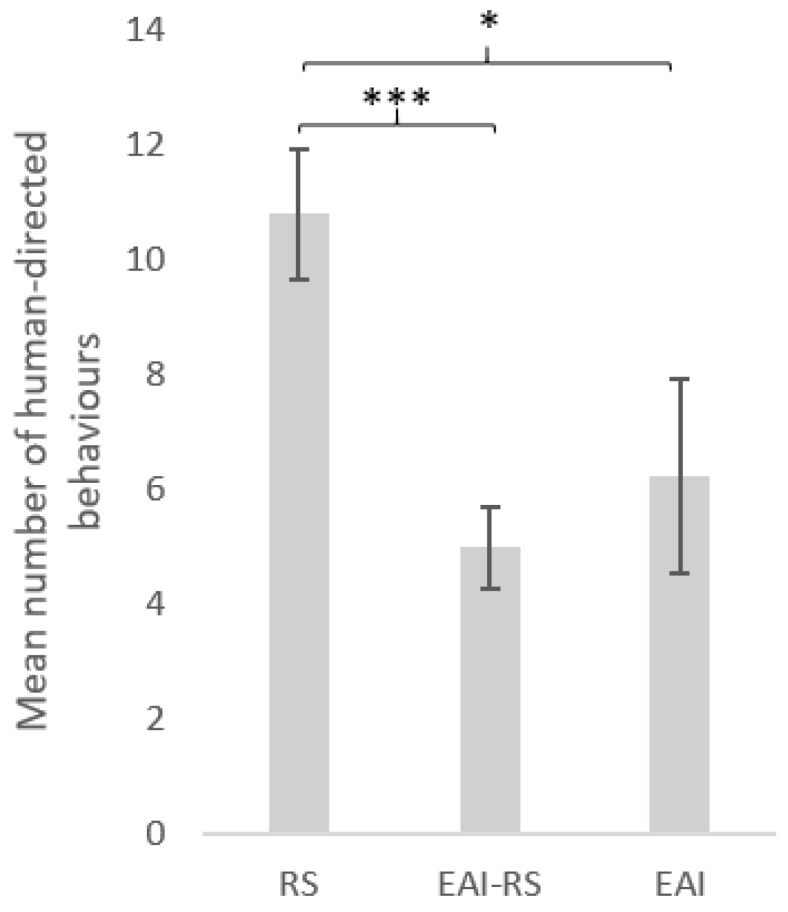
Behaviour differences of horses during the motionless person test according to the horses’ usual activity. Numbers (mean ± SE) of human-directed behaviours according to the horses’ usual activity. GLM negative binomial. RS = riding school horses (N = 95), EAI = assisted-intervention horses (N = 17), EAI-RS = horse with mixed activity (N = 60). ***: *p* < 0.001, *: *p* < 0.05.

**Figure 4:**
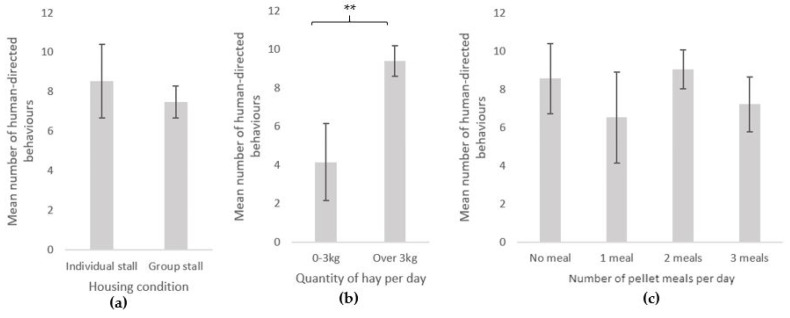
Human-directed behaviours (mean ± SE) during the motionless person test according to equids’ conditions of life parameters: (**a**) housing condition (individual stall: N = 134, group stall: N = 38; (**b**) hay quantity per day (0–3 kg: N = 35, Over 3 kg: N = 137); (**c**) Number of pellet meals per day (No meal: N = 39, 1 meal: N = 17, 2 meals: N = 81 , 3 meals: N = 35). GLM negative binomial. **: *p* < 0.01.

Type of activity was the most important factor (Figure 3). RS equids exhibited more interactive behaviours towards the experimenter (mean = 10.8 ± 1.1) than EAI (mean ± SE = 6.2 ± 1.6) (GLMnb: estimate = 0.58, z-value = 2.06, p-value = 0.039) or EAI-RS (mean ± SE = 5.0 ± 0.7) (GLMnb: estimate = 0.722, z-value = 4. 28, *p*-value < 0.001) equids, but the number of interactive behaviours did not differ significantly between EAI and EAI-RS equids (GLMnb: estimate = 0.15, z-value = 0. 49, *p*-value = 0.632). The second most important factor was the quantity of hay (Figure 4): equids receiving more than 3kgs hay per day produced more interactive behaviours (mean = 9.4 ± 0.8) than equids having less hay (mean = 4.1 ± 1.7) (GLMnb: estimate = 0.52, z-value = 2.47, *p*-value = 0.014).

**Figure 5:**
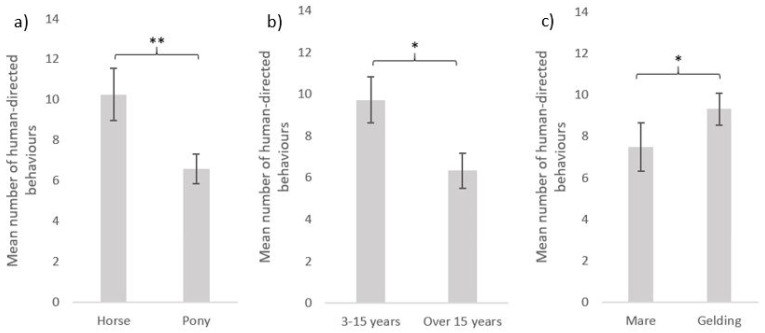
Human-directed behaviours (mean ± SE) during the motionless person test according to equids’ characteristics: (**a**) equine type (horses: N = 81, ponies: N = 91); (**b**) age (3–15 years old: N = 101, over 15 years old: N = 71); (**c**) sex (mare: N = 93, gelding: N = 79). GLM negative binomial. **: *p* < 0.01, *: *p* < 0.05.

The equids’ individual characteristics (type, age and sex) also influenced their interactions with humans (Figure 5). Horses performed more behaviours (mean = 10.3 ± 1.1) than ponies (mean = 6.6 ± 1.0) towards the experimenter (GLMnb: estimate = 0.42, z-value = 2.60, *p*-value = 0.009). Younger equids were more interactive: equids under 15 years performed more interactive behaviours than equids over 15 years of age (mean = 6.3 ± 0.9) towards the experimenter (mean = 9.7 ± 1.1) (GLMnb: estimate = 0.35, z-value = 2.15, *p*-value = 0.032). Geldings (mean = 9.3 ± 1.2) performed more interactive behaviours than mares (mean = 7.5 ± 1.0) towards the experimenter (mean = 10.6 ± 1.2 and 7.7 ± 1.0, respectively; GLMnb: estimate = 0.33, z-value = 2.14, *p*-value = 0.032). 

Distribution of sex and type of equid (pony or horses) were comparable among the three groups (Sex: khi ²: X-squared = 0.25, df = 2, *p*-value = 0.880, type: khi ²: X-squared = 4.18, df = 2, *p*-value = 0.124), but equids used for EAI were overall older (over 15 years) than RS equids (khi ²: X-squared = 5.85, df = 1, *p*-value = 0.016) and tended to be older than EAI-RS (khi ²: X-squared = 3.36, df = 1, *p*-value = 0.067).

Since EAI equids were on average older than RS equids, we performed further statistical tests to test whether age may have influenced the major impact of activities on the behaviours of equids during the MP test. However, within age–class comparisons of the number of behaviours performed by equids during the test confirmed this significant impact of activity (young: Kruskal–Wallis chi-squared = 8.47, df = 2, *p*-value = 0.015; old: Kruskal–Wallis chi-squared = 8.10, df = 2, *p*-value = 0.017). RS tended to perform more interactive behaviours than EAI for older equids (W = 276, *p*-value = 0.073) and differed significantly from EAI-RS for both older (W = 598, *p*-value = 0.007) and younger (W = 1445.5, *p*-value = 0.004) equids. No difference was found between EAI and EAI-RS for either older (W = 142, *p*-value = 0.805) or younger (W = 83, *p*-value = 0.869) equids. 

## 4. Discussion

The results of this study, in which a large sample of riding school and EAI equids underwent the same standardised human–horse relationship test, showed that even though subjects performed overall more positive than negative behaviours towards the experimenter, clear differences appeared according to activity (EAI vs. RS), feeding (hay quantity per day), and equids’ intrinsic characteristics (sex, age, and type). The riding school equids performed more positive behaviours but, most of all, performed, in total, more human-directed behaviours during a test than equids involved in EAI, even if this was only part of their working time. In other words, EAI subjects appeared less interested in humans, i.e., less interactive. Moreover, equids receiving small quantities of hay per day, ponies, older equids, and females were also less interactive than equids with more hay per day, horses, geldings, and younger animals. However, no significant interaction was found between these factors. 

### 4.1. The Valence of Horses’ Behaviours towards Humans

Overall, 30% of our study population displayed at least one negative behaviour, 80% at least one positive behaviour, and 10% did not show any human-directed behaviour; this result was close to the results of some other studies on riding horses using tests with no forced contact, e.g., motionless person test: 60% positive and 15% negative [45]. However, there are large discrepancies in the valence of horses’ reactions during human–horse relationship (HHR) tests, ranging from almost no negative reaction to a majority of negative behaviours [71]. Factors influencing reaction discrepancies include welfare state [26,72,73], feeding conditions [31], health status [74], and working conditions [56,57,58,59], as well as the type of test. Fureix et al. [45] showed that the valence of the reactions in an MP test may be less predictive of the valence of a horse’s reactions, especially when positive, than in other more ‘intrusive’ tests such as approaching or touching the animal, a finding also reported by Burn et al. [28]. Moreover, the relatively high prevalence of positive behaviours in our study may also be due to the fact that we included here measures of positive gazes, which have not been considered in previous studies [45,56] or at least not in the same way [39]. This could be a further interesting measure for future studies. Therefore, the results of our study in terms of valence have to be interpreted with caution even though the finding that RS equines showed more positive behaviours than EAI animals is intriguing and would deserve further consideration.

### 4.2. On the Meaning of Unresponsiveness

In the present study, animals were free to show their interest in humans and their motivation to interact. The lower proportion of interactive responses to the test of EAI equids is remarkable. Unresponsiveness to a human approach has been interpreted as ‘neutral’ by [75,76], whereas it is considered a welfare problem in other studies where unreactive horses were more at risk of presenting skin lesions, low body-condition scores, abnormal mucous membrane colours, or abnormal gaits [28,30,57,77]. Other studies showed that the more horses are depressed, the more they are unresponsive to a human approach [28,29]. 

Popescu and Diugan [59] reported that unresponsiveness changed into negative behaviours when the human approach became more invasive, suggesting that indifference may be a ‘mild’ version or another version of negative valence, expressed when free to initiate the interaction or not. Indeed, in different studies using a motionless person test, the equids produced mostly either positive behaviours and visual attention towards the experimenter or avoidance/indifference but rarely clear negative behaviours [46,61,78,79], contrary to observations performed with more invasive tests [45]. 

### 4.3. Housing and Feeding Conditions 

Contrary to our expectations, we found no significant impact of single versus group housing on the results in the MP test. This is surprising as different studies have shown that social restrictions are deleterious for equids’ welfare [80,81,82] and the human–horse interactions [83,84]. Visser [85], for example, found that horses housed in individual stalls developed more stereotypic behaviours than horses kept in pairs. Group-housing in stalls, contrary to group-housing in larger spaces, may, however, induce tensions due to space limitations and group composition, which may counterbalance the positive effect of social contact. Further studies are needed on this aspect, but overall, studies converge to show that permanent stall housing is not appropriate and may alter equids’ behaviour, in particular during training and work sessions [32,86] and during tests [77]. 

Feeding modalities, in terms of hay availability, appeared, on the contrary, to have an important impact in the present study. Permanent access to roughage allows equids to express a more normal time budget, and many studies have shown its importance for horse welfare [13,17,81,82] and to prevent the emergence of pathologies such as gastric ulcers or colitis [16,87,88]. Horses allowed to graze or feed on roughage all day long appear to show less aggressiveness towards conspecifics [17,89] and to express more positive behaviours towards humans [31]. In the present study, welfare indicators were not collected, and therefore, we cannot definitely conclude on a possible link between welfare state and equids’ reactions to the MP test, but we can hypothesise that a better welfare state could explain the differences we found here, suggesting further that being interactive with humans may reflect a more positive state. Indeed, Lansade et al. [33] showed that equids in more appropriate living conditions were more interactive during a motionless person test than equids in an impoverished environment.

### 4.4. Individual Behavioural Characteristics

Some researchers suggest that horses’ reactions to humans are temperament traits [34], and individual differences have been demonstrated [44]. These individual differences are partly related to genetic factors such as breed or sire. At 6 months, foals’ facilitation of human contact by their dam is modulated by their sire origin [41]. A study of adult horses all living in the same facility showed that French saddlebreds were more friendly in a sudden approach test than Angloarabs, whereas Thoroughbreds were more indifferent [39]. Cold-blooded horses were less interactive than warm-blooded ones in a motionless test [90]. Our study equids came from too many breeds and too many were unregistered to test for a breed or sire effect, but ponies appeared less reactive towards humans than horses. This is in line with the study by Henriksson et al. [40], although Schrimpf et al. [43] and Maros et al. [91] found no differences or even opposite results. However, their results were obtained in tests not involving human relationships. These mitigated results may be due also to the side-effects of selection for other traits or to different equid managements. 

We detected other individual characteristics that modulated horses’ reactions to humans. The sex differences found here, with geldings being more interactive, have not been recorded in other studies using the same test [36,37,38,78]. Popescu et al. [92] reported that stallions appeared to be more indifferent and mares more aggressive, but since these animals had different housing conditions, it is difficult to draw definitive conclusions. Similar results were reported by Wulf et al. [93] for yearling horses, but males and females had different handlers in this study and, as Fureix et al. [45] showed, daily interactions with their caretaker can impact a human–horse relationship. Overall, studies of the social behaviour of domestic horses report that geldings are more interactive socially [94,95]. Although to our knowledge it is still not clear, one hypothesis would be that this extends to the interspecific relationship context. 

Younger horses are generally more socially interactive [96] and explore more [97], which may explain the age differences found here that are similar to those found by Burn et al. [28] and Popescu and Diugan [30]. There was no indication that older horses had more health problems (caretakers’ reports, direct observations), and earlier studies have shown, for example, that the prevalence and extent of back disorders were not correlated with age (e.g., review in [25]). 

### 4.5. EAI as a Working Activity

As mentioned above, working conditions, when inappropriate, can have a negative impact on horses, such as an increased prevalence of abnormal behaviours, higher emotionality, or physical health problems [18,20,21,25,98,99,100,101]. Positive training leads on the contrary to improved HHR [46,61].

While research studies on EAI mention no particular problem with this activity [48,51,52,54,55], the questions of the impact of the characteristics of the clients involved and of the modalities of interventions still remain [10,11]. Indeed, during EAI, horses are often requested to interact with persons who may have unpredictable behaviours [51], attention problems, be aggressive or irritable [10], and are in a negative mood [52]. In other cases, persons with a balance problem [10] can have poor stability and an asymmetrical position on the saddle that can cause lameness or back pain in horses [102,103]. As there are discrepancies in the methods and indicators used for assessing how horses react during the EAI sessions, further studies are needed in order to elucidate whether there may be some aspects of the EAI that are more difficult to deal with for the horses than others.

## 5. Conclusions 

Overall, it is not possible at this stage to know why the EAI equids in our study were less interactive with the experimenter in the test, i.e., whether they are more depressed due to their activity, whether they may have been trained for not reacting in any human-related situation, or if they had been chosen for EAI because of their overall low reactivity, including their reactions to humans (e.g., in order to decrease the risks for the clients of EAI). However, studies aiming at testing EAI equids’ temperament have not led to any conclusive difference with other working horses [50,104].

Further studies are needed that will include different types of tests, welfare assessments, and observations during EAI sessions (Lerch et al. in preparation). In any case, since it is well known that positive training can lead to improved human–horse relationships [46,61], it could be interesting to associate EAI with positive reinforcement, as recommended by the International Association of Human–Animal Interaction Organisations [1], in order to have more positively interactive and attentive horses and, at the same time, improve their welfare. 

## Figures and Tables

**Table 1:** Subjects’ characteristics.

Centre	Country	Date	Number of Equids According to								Mean Age (Years) ±SE
			Total	Activity			Sex		Type		
				EAI	EAI-RS	RS	Mares	Geldings	Horses	Ponies	
Centre1	France	03/2010	9	0	4	5	6	3	1	8	14.4 ± 0.4
Centre2	France	01/2010	6	0	0	6	2	4	2	4	11.0 ± 1.6
Centre3	France	09/2010	11	0	0	11	3	8	7	4	14.7 ± 1.5
Centre4	France	04/2010	10	0	7	3	6	4	5	5	14.6 ± 1.6
Centre5	France	11/2009 & 09/2010	18	0	0	18	10	8	10	8	14.1 ± 1.4
Centre6	Italy	11/2017	9	9	0	0	5	4	9	0	21.6 ± 2.2
Centre7	Italy	11/2017	2	0	2	0	2	0	0	2	19.0 ± 1
Centre8	Italy	12/2017	7	0	7	0	3	4	1	6	14.3 ± 1.5
Centre9	Italy	11/2017	6	2	0	4	6	0	2	4	21.3 ± 1.3
Centre10	France	01/2019	34	0	0	34	18	16	15	19	11.9 ± 0.8
Centre11	France	02/2019	27	2	17	8	20	7	15	12	13.2 ± 1.2
Centre12	Ireland	04/2019	33	4	23	6	12	21	14	19	14.8 ± 0.8
All centres			172	17	60	95	93	79	81	91	14.3 ± 0.4

RS = riding school lessons horses, EAI = assisted-intervention horses, EAI-RS = horses with mixed activity.

**Table 2:** List of behaviours recorded during the motionless person test (adapted from Fureix et al. [45]).

Behaviours		Description
Positive (ears forwards and towards the experimenter)	Gazes	Equid uses its binocular vision field to look at the experimenter for at least 1 second.
	Approaching	Equid walks towards the experimenter, with ears and gaze oriented towards the experimenter.
	Sniffing	Equid sniffs the experimenter.
	Licking	Equid sticks out its tongue and contacts the experimenter.
	Nibbling	With jaws closed, the upper lip moves upwards and downwards over the experimenter, typically without dental contact with the object.
Invasive contacts	Biting clothes	Equid takes part of the experimenter’s clothes into its mouth with its teeth and pulls them.
	Head rubbing	Equid rubs its head on the experimenter.
	Pushing with head	Equid pushes the experimenter with its head.
	Jostling	Equid jostles the experimenter with its body.
Negative (ears backwards)	Threatening gazes	Equid looks at the experimenter.
	Threatening approaches	Equid approaches threateningly.
	Threatening sniffs	Equid sniffs the experimenter.
	Threatening nibbles	With jaws closed, moves its upper lip upwards and downwards over the experimenter, typically without dental contact with the object.
	Threatening to bite	Equid looks at the experimenter with ears laid back and showing its teeth.
	Threatening to kick	Equid points its rump towards the experimenter and can lift a leg towards the experimenter but without making contact.
	Kicking	Equid kicks the experimenter.
	Moving away	Equid moves away from the experimenter.
	Turning its back	Equid turns its back to the experimenter.
Others (ears asymmetrical or sidewards)	Other gazes	Equid’s neck is oriented towards the experimenter, and it uses its binocular vision to look at the experimenter for at least 1 second.
	Approaching	Equid walks towards the experimenter, with gaze oriented towards the experimenter.
	Sniffing	Equid sniffs the experimenter.
	Nibbling	With jaws closed, moves its upper lip upwards and downwards over the experimenter, typically without dental contact with the object.

## Data Availability

The data generated and analysed during the current study are available from the corresponding author on reasonable request.
